# A Review of the Botany, Phytochemistry, Pharmacology and Toxicology of Rubiae Radix *et* Rhizoma

**DOI:** 10.3390/molecules21121747

**Published:** 2016-12-20

**Authors:** Mingqiu Shan, Sheng Yu, Hui Yan, Peidong Chen, Li Zhang, Anwei Ding

**Affiliations:** Jiangsu Collaborative Innovation Center of Chinese Medicinal Resources Industrialization, National and Local Collaborative Engineering Center of Chinese Medicinal Resources Industrialization and Formulae Innovative Medicine, Nanjing University of Chinese Medicine, Nanjing 210023, China; yusheng1219@163.com (S.Y.); glory-yan@163.com (H.Y.); chenpeidong1970@163.com (P.C.); zhangliguanxiong@163.com (L.Z.)

**Keywords:** botany, pharmacology, phytochemistry, review, Rubiae Radix *et* Rhizoma, toxicology

## Abstract

*Rubia cordifolia* Linn (Rubiaceae) is a climbing perennial herbal plant, which is widely distributed in China and India. Its root and rhizome, Rubiae Radix *et* Rhizoma (called Qiancao in China and Indian madder in India), is a well known phytomedicine used for hematemesis, epistaxis, flooding, spotting, traumatic bleeding, amenorrhea caused by obstruction, joint impediment pain, swelling and pain caused by injuries from falls. In addition, it is a kind of pigment utilized as a food additive and a dye for wool or fiber. This review mainly concentrates on studies of the botany, phytochemistry, pharmacology and toxicology of this Traditional Chinese Medicine. The phytochemical evidences indicated that over a hundred chemical components have been found and isolated from the medicine, such as anthraquinones, naphthoquinones, triterpenoids, cyclic hexapeptides and others. These components are considered responsible for the various bioactivities of the herbal drug, including anti-oxidation, anti-inflammation, immunomodulation, antitumor, effects on coagulation-fibrinolysis system, neuroprotection and other effects. Additionally, based on these existing results, we also propose some interesting future research directions. Consequently, this review should help us to more comprehensively understand and to more fully utilize the herbal medicine Rubiae Radix *et* Rhizoma.

## 1. Introduction

*Rubia cordifolia* Linn (*R. cordifolia*, *Rubiaceae*) is a climbing perennial herbal plant widely distributed in China and India. As a medicinal plant, it was firstly recorded in a formula with endoconch of *Sepiella maindroni* in “*Huangdi Neijing*”, the most important ancient book in Chinese Traditional Medicine (TCM). Rubiae Radix *et* Rhizoma (RRR), the dried root and rhizome of *R. cordifolia*, is a famous TCM used for thousands of years. The medicinal part of *R. cordifolia* was specificly presented for the first time in “*Shennong Bencaojing*”, another classic work on plants and their uses. Since then, this phytomedicine has been employed for hematemesis, epistaxis, flooding, spotting, traumatic bleeding, amenorrhea caused by obstruction, joint impediment pain, swelling and pain caused by injuries from falls [1]. With its excellent activity to cool blood, eliminate stasis, stop bleeding and unblock meridians, it has been considered in “*Bencao Gangmu*” and other ancient medical books as an important herbal drug for curing the syndromes caused by blood heat. Thus, RRR is still listed in the *Chinese Pharmacopeia* [1]. During the past decades, more than one hundred chemical compounds such as anthraquinones, naphthoquinones, cyclic hexapeptides, terpenoids, polysaccharides and other compounds have been found in RRR, isolated and identified [2,3,4,5,6,7,8,9,10]. The range of its pharmacological activity also has been widened, covering antioxidation, neuroprotection, anti-inflammation, antitumor, and immunomodulation effects, etc [3,11,12,13,14,15,16,17].

Additionally, *R. cordifolia* is a well-known source of red plant dye for clothes and food items with a long history of use in China and India [18,19,20,21,22]. The earliest record of this was in “*Shijing*”, an ancient book about over 2500 years ago. In China, RRR has been listed by the Ministry of Health as one of the substances that can be used in dietary supplements.

With the developments of science and technology and the introduction of many advanced experimental methods and instruments during the recent decades, a large number of studies have been performed on many aspects of RRR and a lot of achievements have been made, which were reported in many literatures. So, to summarize these studies and findings, we aim with this paper to provide a comprehensive and up-to-date review of Rubiae Radix *et* Rhizoma that covers the studies of its botany, phytochemistry, pharmacology and toxicology.

## 2. Botany

According to the *Chinese Pharmacopeia* [1], *R. cordifolia* Linn is the sole and authentic plant source of Rubiae Radix *et* Rhizoma. At present, *R. cordifolia* L. *varstenophylla* Franch, *R. cordifolia* L. *varherbacea* Chun *et* How, *R. cordifolia* L. *varpratensis* Maxim and *R. cordifolia* L. var*mollis* Chun *et* How are considered as the indigenous varieties of *R. cordifolia* Linn [23]. In China, it is widely distributed in most regions, including the provinces of Shaanxi, Henan, Anhui, Hebei, Shandong, Hubei, Jiangsu, Zhejiang, and so on. Among them, Weinan Shannxi and Songxian Henan are the most fundamental production areas, famous for their high production volume and quality [24]. In some areas of China, *R. schumanniana* pritz., *R. yunnanensis* Diels, *R. membranacea* Diels, *R. tinctorum* L. and so on are used as folk medicines rather than *R. cordifolia* Linn. Nevertheless, neither of them is the authentic one [25].

**Figure 1:**
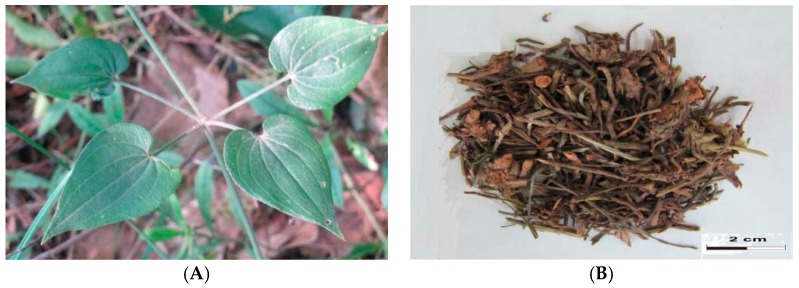
*Rubia cordifolia* Linn (**A**) and Rubiae Radix *et* Rhizoma (**B**).

*R. cordifolia* is a climbing perennial herbal plant. The roots, which cluster in the soil, are aubergine or orange-red. The elongating and rough stems slightly lignify at the base. The branches are four-edge shaped and there are some anatropousspinules on the edges. The papery leaves are ovate or ovate-lanceolate and 2–6 cm long, 1–3 cm wide [24]. Sometimes there are sparse bristles on the rough-surfaced leaves. The cymes are in the axils or on the tops and are in the shapes of large and loose cones. The subsphaeroidal fruits with smooth surfaces, about 5 cm in diameter, are black or black-purple when ripening. Growing at the altitude of 570–1800 m, *R. cordifolia* is found along the roadside or riverside, on hillsides and in valleys [23]. Generally, the root and rhizome are collected in spring or autumn in the third or fourth year after cultivation [25]. Figure 1 shows *R. cordifolia* and Rubiae Radix *et* Rhizoma.

## 3. Phytochemistry

More than a hundred components, which belong to anthraquinones, naphthoquinones, cyclic hexapeptides, terpenoids and other classes of compounds, have been found and isolated from RRR. Some of them have been proved responsible for the pharmacological activities in many studies. The compounds reported in the literature are listed in Table 1.

### 3.1. Anthraquinones

**Figure 2:**
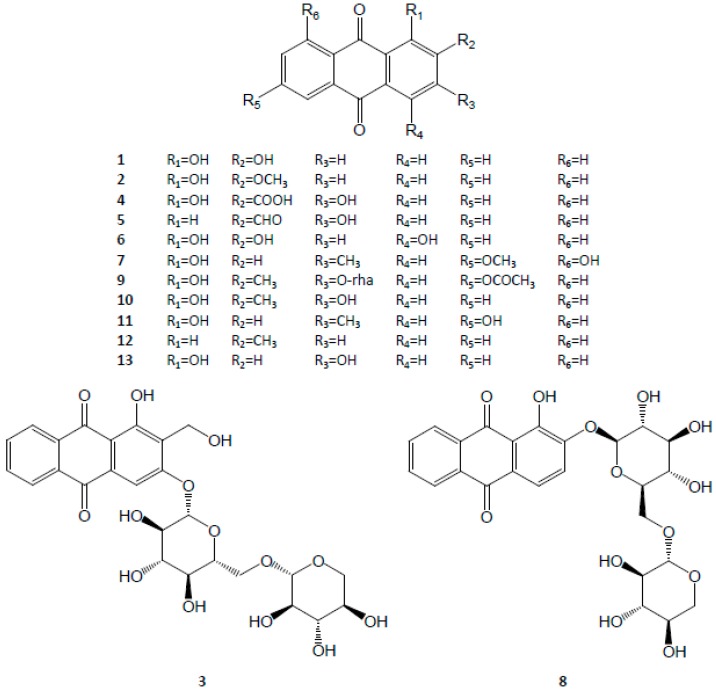
Structures of anthraquinones in Rubiae Radix *et* Rhizoma.

Anthraquinone is a well-known category of phytochemicals. Alizarin, munjistin, purpurin, rubiadin, tectoquinone and xanthopurpurin are the common ones. Among them, purpurin is one of the two chemical markers that serve to evaluate the quality of the herbal medicine in the *Chinese Pharmacopeia* [1]. In pharmacological studies, it has been proved to have anti-angiogenic [67], and anti-oxidant [68,69] functions. Besides, these anthraquinonesare often found combined with glucoside, xylosyl-(1→6)-glucoside (primeverose) or rhamnosyl-(1→2)-glucoside to form the corresponding anthraquinone glycosides. The structures are shown in Figure 2.

### 3.2. Naphthoquinones

**Figure 3:**
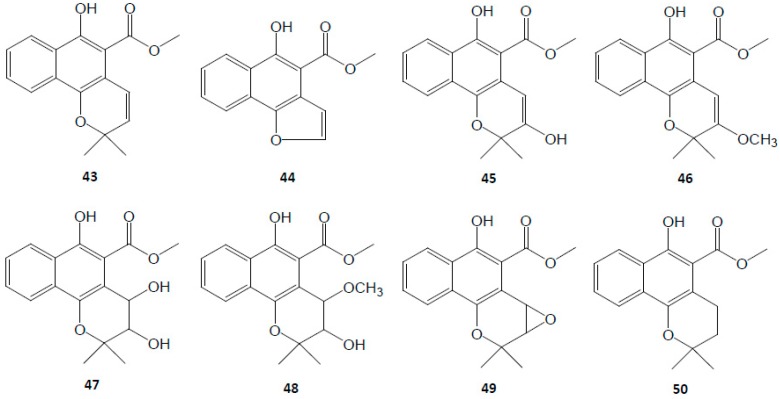
Structures of naphthoquinones in Rubiae Radix *et* Rhizoma.

Naphthoquinone is another category of phytochemicals in RRR. Mollugin is the representative on, reported to have anti-cancer [70,71], anti-inflammatory [17,72,73], and neuroprotective activities [72]. With a content of 10^−3^ g/g level or even 10^−2^ g/g level in RRR, it is designated as the other chemical marker by the *Chinese Pharmacopeia* [1]. Meanwhile, some derivatives of mollugin were isolated, too. The structures are shown in Figure 3.

Besides the naphthoquinone monomers above, three naphthohydroquinone dimers were also isolated, which were chemically named as 6-hydroxy-2-(5-hydroxy-4-methoxycarbonylnaphtho-[1,2-*b*]furan-2-yl)-2-methyl-3,4-dihydro-2*H*-benzo[*h*]-chromene-5-carboxylic acid methyl ester, rubioncolin B and 5-hydroxy-2-[7-hydroxy-4-(1-hydroxy-1-methylethyl)-2-methyl-6-oxo-2,3,3a,6-tetrahydro-4*H*-1,5-dioxa-benzo-[de]anthracen-2-yl]-naphtho[1,2-*b*]furan-4-carboxylic acid methyl ester.

### 3.3. Cyclic Hexapeptides

**Figure 4:**
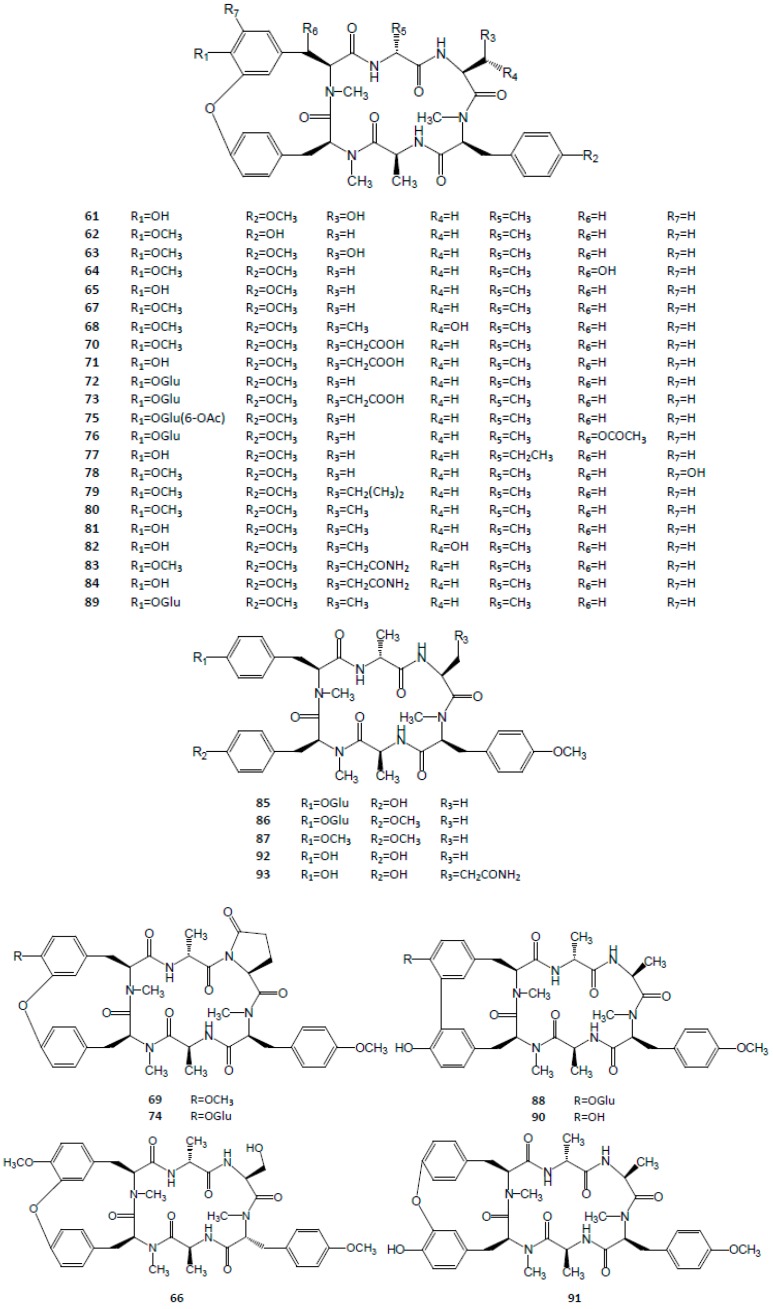
Structures of cyclic hexapeptides in Rubiae Radix *et* Rhizoma.

Some Japanese researchers were the first to isolate four cyclic hexapeptides from *R. cordifolia* and *R. akane* while screening for anticancer compounds in phytomedicines. Up to now, twenty four cyclic hexapeptides have been found and isolated from RRR, which were named as RA I-XXIV. Each RA contains both 18-member ring and 14-member ring systems, which consist of some amino acids including *N*-methyl-*O*-methyl-l-tyrosine, pyroglutamic acid, l-alanine, d-alanine, etc. Among these RAs, RA-V and RA-VII were the dominating two, found at levels of nearly 100 μg/g, while the others represent less than 1 μg/g in RRR. Furthermore, in recent years, some analogues or precursors of RAs were also isolated from RRR, such as neo-RA-V, allo-RA-V, rubicordin A-C, *O*-seco-RA-V, *O*-seco-RA-XXIV, and so on. The structures are shown in Figure 4.

### 3.4. Triterpenoids

Among phytomedicines, oleanolic acid and ursolic acid are common triterpenoids. The two compounds also have been found and isolated in RRR. Rubiprasin A-C, rubiarbonol A and B, rubicoumaric acid and rubifolic acid and other triterpenoids were isolated, too.

### 3.5. Other Compounds

Other than anthraquinones, naphthoquinones, cyclic hexapeptides and triterpenoids, phytochemistry researchers have found and isolated many other chemical compounds from RRR, including some organic acids, polysaccharides, rubilactone, rubiasin A-C, β-sitostenone, β-sitosterol, 5-methoxygeniposidic acid, 6-methoxygeniposidic acid, and so on.

## 4. Pharmacology

In many early pharmacological studies, RRR has been proved to have various functions, such as radioprotective [74], antidiabetic [75], hepatoprotective [76], antitumor [48,50,51,52,54,77], antiplatelet [78] effects, and so on [44], which were reported in previous reviews [79,80]. However, in recent years, some new activities and mechanisms were found that had not been previously reviewed.

### 4.1. Effects on the Coagulation-Fibrinolysis System

In TCM, RRR is well known for its activity of cooling blood, eliminating stasis, stopping bleeding and unblocking meridians [1]. In our previous study, we produced a rat model with blood stasis using a subcutaneous injection of adrenaline plus an ice water bath. After the model rats were treated with RRR, whole blood viscosity and plasma viscosity decreased and content of fibrinogen increased. In addition, shortened prothrombin time, prolonged thrombin time and activated partial thromboplastin time in the model rats were rectified after treatment with RRR. Furthermore, compared to model rats, RRR treatment also downregulated thromboxane B_2_ (TXB_2_) levels and upregulated 6-keto-PGF1α levels [81]. Evidence of a study on the coagulation-fibrinolytic system of normal rats revealed that RRR decotion elevated the activity of tissue plasminogen activator (t-PA) [82].All these findings prove the effects of RRR on the blood system.

As we know, plasma hyaluronan-binding protein (PHBP) is a serine protease able to activate coagulation factor VII and prourokinase and to circulate as a single-chain form. With spermidine or heparin, it is autoproteolytically converted into an active two-chain form. In a screening investigation, purpurin was identified as a specific inhibitor of spermidine-induced autoactivation of PHBP [83].

### 4.2. Antitumor

RRR methanol extract (RRRME) was found to inhibit human laryngeal carcinoma cell (HEp-2 cell) proliferation and lactate dehydrogenase (LDH) release, to decrease reduced glutathione (GSH), glutathione S-transferase (GST) and protein levels, and to increase lipid peroxidation (LPO) in a dose-dependent manner. Further fluorescent microscopy and transmission electron microscopy confirmed this apoptotic effect [15].

Many studies have demonstrated that the cyclichexapeptides were the bioactive components responsible for the antitumor activity of RRR. The majority of the RA series compounds showed cytotoxicity against many cancer cells, including P-388 leukaemia cells [64], SGC-7901 human gastric adenocarcinoma cells, A-549 human non-small cell lung carcinoma cells, and Hela (human cervical carcinoma) cells [3].

Some small molecule compounds in RRR are also important antitumor compounds. As an Ames assay showed, alizarin was found effective in reducing his+ revertants induced by 4-nitro-*o*-phenylenediamine and 2-aminofluorene. Comet assay results indicated that DNA damage induced by H_2_O_2_ and 4-nitroquinoline-1-oxide could be reduced by anthraquinone too [84]. Mollugin was also proved to potentiate autophagic activity, induce growth inhibition and apoptosis of HN4 human oral cancer cells and SK-BR-3 breast cancer cells. It could upregulate the expression of mammalian target of rapamycin (mTOR), downregulate fatty acid synthase(FAS) gene expression and activate nuclear factor-E2-related factor 2 (Nrf2) with heme oxygenase-1 (HO-1)via some signaling pathways, such as phosphatidylinositol 3-kinase/protein kinase B (PI3K/AKT) and extracellular signal–regulated kinases (ERK). This naphthoquinone could also inhibit the activation of nuclear factor-κB (NF-κB) and NF-κB-dependent gene products involving antiapoptosis (Bcl-2 and Bcl-xl), invasion (MMP-9 and ICAM-1), and angiogenesis (FGF-2 and VEGF) [70,71,85]. 1-Hydroxy-2methylanthraquinone was another compound from RRR exhibiting cytotoxic effects on A375 malignant skin melanoma cells [86].

### 4.3. Immunomodulation

RRR ethanol extract (RRREE) was found to have protective effect against immunosuppression of Swiss albino mice induced by lead nitrate. This immunomodulation was considered associated to the increasing of macrophagocyte number and phagocyticindex, immunoglobulin levels and plaque-forming cell number [13]. In the serum of most peanut-allergy patients, there were increased levels of immunoglobulin E (IgE). After animals of a peanut-anaphylaxis mice model were treated with RRR aqueous extract, peanut-triggered anaphylactic reactions and plasma histamine levels decreased significantly, as did IgE production by a human B-cell line [14].

### 4.4. Anti-Inflammation

Pharmacological evidences showed that RRREE was able to ameliorate the lead nitrate-induced oxidative damage by improving the activities of superoxidedismutase (SOD) and catalase (CAT), increasing the content of GSH, and suppressing LPO [13]. In another study of indomethacin-induced enterocolitis in rats, some acute intestinal inflammation effects such as bowel wall thickening, mesenteric haemorrhage, mesentery adhesion and multiple mucosal ulcers of small intestine and colon emerged. Serum LDH activity increased, too. These disorders and changes were ameliorated after RRREE-treatment [87].

After RAW 264.7 macrophages were stimulated by LPS, the contents of NO, inducible nitric oxide synthase (iNOS), interleukin-1β (IL-1β) and interleukin-6 (IL-6) increased significantly. However, mollugin-incubation brought some reduction of these inflammatory mediators [17]. In a screening investigation of some phytochemicals, physcion and 1-hydroxy-2methylanthraquinone showed amelioration on the damage to mouse peritoneal macrophages induced by lipo-polysaccharide (LPS) and interferon-γ (IFN-γ), which was mediated through inhibition of iNOS protein expression and reduction of NO content [86].

### 4.5. Neuroprotection

In a study of reserpine-induced orofacial dyskinesia, RRRME-treatment was able to significantly inhibit vacuous chewing movements, tongue protrusions, orofacial bursts, catalepsy and to increase locomotion and rearing in an open field test. Meanwhile, bioassay results revealed that RRRME-treatment increased the levels of SOD, CAT, GSH, inhibited LPO and elevated dopamine levels in the forebrain region, compared with the model. These findings demonstrated that the neuroprotection of RRRME was related to its antioxidant activity [88].

Mollugin was considered as a neuroprotective agent for glutamate-induced neurotoxicityin the mousehippocampal HT22 cell line. The neuroprotection may be mediated by the effects on suppression of pro-inflammatory mediators, up-regulation of the expression ofHO-1 and the activity of HO, nuclear accumulation of Nrf2 and activation of p38 mitogen-activated protein kinase (MAPK) pathway [72]. In the T-REx293human embryonic kidney cell line, Aβ(42)-EGFP (enhanced green fluorescent protein) plays a key role in Alzheimer’s disease. Abnormal accumulation of Aβ(42)-EGFP would lead to apoptosis of this cell line. With treatment by RRR polysaccharides, Aβ(42)-EGFP accumulation decreased and cell activity was restored dramatically. Moreover, RRR polysaccharides inhibited cytotoxicity of Aβ(42)-EGFP, which may be mediated by potentiated degradation of proteosome [11].

### 4.6. Antioxidation

Results of studies on the gastroprotective effect on aspirin plus pylorus-ligated ulcer indicated that RRRME and its chloroform fraction brought notable decreases of ulcer index, total acidity, protein, pepsin content of the gastric fluid and increase of the mucin content. Among the key antioxidant parameters, after treatment with RRRME or its chloroform fraction, were a significant reduction in LPO and elevations in CAT, SOD, and GSH. It was concluded that the protective mechanism could perhaps be partly attributed to the effects of the herbal medicine on oxidative stress [12].

In *N*-nitrosodiethylamine-induced hepatocellular carcinoma rats, the activities of serum marker enzymes including aspartate transaminase (AST), alanine aminotransferase (ALT), alkaline phosphatase (ALP) and LDH with the levels of LPO and hydroxyl radicals in liver increased significantly. The opposite trends were seen with the activities of the antioxidants including SOD, CAT, GSH, GST, glutathione peroxidase (GPx) in liver and the levels of mitochondrial enzymes like isocitrate dehydrogenase (ICDH), succinate dehydrogenase (SDH), α-ketoglutarate dehydrogenase (α-KGDH) and respiratory chain enzymes like nicotinamide adenine dinucleotide (NADH) dehydrogenase and cytochrome c oxidase. However, all these changes in the model group were markedly and dose-dependently ameliorated by treatment of RRRME, which indicated RRR perhaps could be used as an antioxidant for the treatment of some cancers [16]. Structure-radical scavenging activity relationship results demonstrated that hydroxyl groups on the benzene rings were essential to the radical scavenging function of hydroxylanthraquinone [89].

### 4.7. Other Pharmacological Functions

Besides the effects above, RRR have exhibited good effects in studies of anti-urolithiasis, anti-psoriasis, anti-nephrotoxicity, estrogenic and progestational activity, and so on [47,90,91,92,93,94,95] (Table 2).

## 5. Toxicology

Though some extracts or compounds from RRR have shown antitumor effects, rubiadin was reported to display carcinogenic potential. In the outer medulla, cytoplasmic swelling with basophilic changes and karyomegaly were observed in male F344 rats fed with rubiadin for one week whereas 26-week oral administration of the component induced atypical tubules, putative pre-neoplastic lesions, and karyomegaly. The results indicated that rubiadin may be a potent carcinogenic ingredient that targeted the proximal tubule cells in the outer medulla [96]. Rubiadin was also considered as both initiator and promoter of carcinogenicity targeting kidney, liver and large intestine [97]. In madder pigment, alizarin, purpurin and 1-hydroxyanthraquinone were found to have similar effects as ethidium bromide, a typical DNA intercalator. They exhibited potential genotoxicity by implanting into the DNA of *Escherichia coli*, blocking gene expression and inducing cell death [98].

## 6. Conclusions and Remarks on Future Work

As one of the earliest TCMs used in the clinic, RRR has shown various actions on many syndromes and indications for over 2000 years. In this paper, we have provided a review of RRR focused on the fields of botany, phytochemistry, pharmacology and toxicology based on the data and results collected from a large amount of research studies.

Rubidate is a synthetic derivative of ruberythric acid. It was documented to have the property of increasing leukocyte levels in peripheral blood [99,100] and developed into a medicine for the treatment of the leucopenia [101] in China in the 1980s. At present, over 100 components have been isolated from RRR, including anthraquinones, naphthoquinones, cyclic hexapeptides and triterpenoids, etc. In structure-effect relationship studies, with bioactivity-guided and high-throughput screening methods, other components from RRR or their synthetic derivatives may potentially be found as candidate drugs like rubidate.

In traditional clinical use, RRR has a long history of use in some hemorrhages induced by blood stasis for its activities of stopping bleeding and resolving stasis, which had been proved by some pharmacological experiments [74,75,76]. However, according to modern pharmacology, the two effects are contradictory on some indicators of coagulative and fibrinolytic systems. In a pathological state, stanching would cause stasis and blood-activating would induce hemorrhages, so there still remain some questions to be answered, like what are the active components or fractions responsible for these two seemingly conflicting functions, respectively? How can they act to maintain a healthy state of relative equilibrium rather than pathological state of hemorrhage or stasis?

There is no doubt that RRR is an effective TCM in clinical practice with a long history and plenty of accumulated experiences. Nevertheless, the mutagenicity or carcinogenic potential of the active anthraquinones is a matter of concern [102,103]. At present, there is lack of research and literature on the in vivo metabolism and the metabolites of these components, so in terms of safe medication, the Absorption-Distribution-Metabolism-Excretion-Toxicity (ADMET) of these naturally occurring anthraquinones should be the urgent concern right now.

The present literature review provides a full-scale profile of various aspects of Rubiae Radix *et* Rhizoma and proposes some issues worth investigating in the future. We believe that it will help us to comprehensively understand and more effectively develop this traditional phytomedicine.

## Figures and Tables

**Table 1:** Compounds in Rubiae Radix *et* Rhizoma.

Category	No.	Compound	Molecular Formula	Reference
Anthraquinones	1	Alizarin	C_14_H_8_O_4_	[6,9,26,27,28]
	2	Alizarin 2-methyl ether	C_15_H_10_O_4_	[29]
	3	Lucidinprimeveroside	C_26_H_28_O_14_	[6,9,30]
	4	Munjistin	C_15_H_8_O_6_	[7,31]
	5	Nordamnacanthal	C_15_H_8_O_5_	[32]
	6	Pupurin	C_14_H_8_O_5_	[9,28,31]
	7	Physcion	C_16_H_12_O_5_	[32,33]
	8	Ruberythric acid	C_25_H_26_O_13_	[6,9,28,30]
	9	Rubiacordone A	C_23_H_22_O_10_	[34]
	10	Rubiadin	C_15_H_10_O_4_	[7,9,35]
	11	Soranjidiol	C_15_H_10_O_4_	[29]
	12	Tectoquinone	C_15_H_10_O_2_	[9,36,37,38]
	13	Xanthopurpurin	C_14_H_8_O_4_	[7,9,28,36]
	14	1-Hydroxyanthraquinone	C_14_H_8_O_3_	[30]
	15	1-Hydroxy-2-methylanthraquinone	C_15_H_10_O_3_	[6,7,9,32,36,37]
	16	1,4-Dihydroxy-6-methylanthraquinone	C_15_H_10_O_4_	[32]
	17	1-Hydroxy-2-methoxyanthraquinone	C_15_H_10_O_4_	[35]
	18	1,3-Dimethoxy-2-carboxylanthraquinone	C_17_H_12_O_6_	[35]
	19	1,3,6-Trihydroxy-2-methylanthraquinone	C_15_H_10_O_5_	[6,7,8,26,30,39]
	20	1,4-dihydroxy-2-methylanthraquinone	C_15_H_10_O_4_	[9,36,40]
	21	1,4-Dihydroxy-2,3-dimethylanthraquinone	C_16_H_12_O_4_	[41]
	22	1,5-Dihydroxy-2-methylanthraquinone	C_15_H_10_O_4_	[40]
	23	1,3-Dihydroxy-2-ethoxymethylanthraquinone	C_17_H_14_O_5_	[6,41]
	24	1,4-Dihydroxy-2-methy-5-methoxylanthraquinone	C_16_H_12_O_5_	[9]
	25	1-Acetoxy-3-methoxyanthraquinone	C_17_H_12_O_5_	[29]
	26	1-Hydroxy-3-carbomethoxyanthraquinone	C_16_H_10_O_5_	[36]
	27	1-Hydroxy-2-hydroxymethylanthraquinone	C_15_H_10_O_4_	[8,36]
	28	1-Hydroxy-3-hydroxymethylanthraquinone	C_15_H_10_O_4_	[41]
	29	1-Hydroxy-3-ethylanthraquinone	C_16_H_12_O_3_	[41,42]
	30	1-Hydroxy-2,7-dimethylanthraquinone	C_16_H_12_O_3_	[2]
	31	1,2,4,6-Tetrahydroxyanthraquinone	C_14_H_8_O_6_	[43]
	32	1,2,4-Trihydroxylanthraquinone	C_14_H_8_O_5_	[30]
	33	2-Hydroxy-6-methylanthraquinone	C_15_H_10_O_3_	[2]
	34	2,6-Dihydroxyanthraquinone	C_14_H_8_O_4_	[2]
	35	1,3,6-Trihydroxy-2-methyl-9,10-anthraquinone-3-*O*-β-glucoside	C_21_H_20_O_10_	[7,30]
	36	1,3,6-Trihydroxy-2-methylanthraquinone-3-*O*-α-rhamnosyl-(1→2)-β-glucoside	C_27_H_30_O_14_	[6,7,39]
	37	1,3,6-Trihydroxy-2-methylanthraquinone-3-*O*-(3′-*O*-acetyl)-α-rhamnosyl-(1→2)-β-glucoside	C_29_H_32_O_15_	[7]
	38	1,3,6-Trihydroxy-2-methylanthraquinone-3-*O*-(6′-*O*-acetyl)-α-rhamnosyl-(1→2)-β-glucoside	C_29_H_32_O_15_	[6,7]
	39	1,3,6-Trihydroxy-2-methylanthraquinone-3-*O*-(4′,6′-*O*-diacetyl)-α-rhamnosyl-(1→2)-β-glucoside	C_31_H_34_O_16_	[7]
	40	1,3,6-Trihydroxy-2-methylanthraquinone-3-*O*-(3′,6′-*O*-diacetyl)-α-rhamnosyl-(1→2)-β-glucoside	C_31_H_34_O_16_	[7,39]
	41	1,3,6-Trihydroxy-2-methylanthraquinone-3-*O*-(6′-*O*-acetyl)-α-xylopyranosyl-(1→2)-β-glucoside	C_28_H_30_O_15_	[30]
	42	1,8-Dihydroxy-11,20(15-pentylnaphthaquinonyl) phenanthrene	C_26_H_20_O_4_	[42]
Naphthoquinones	43	Mollugin	C_17_H_16_O_4_	[6,7,9,36,44,45]
	44	Furomollugin	C_14_H_10_O_4_	[29,44,45,46]
	45	2′-Hydroxymollugin	C_17_H_16_O_5_	[8,38]
	46	2′-Methoxymollugin	C_18_H_18_O_5_	[8]
	47	1′,2′-Dihydroxydihydromollugin	C_17_H_18_O_6_	[8]
	48	1′-Methoxy-2′-hydroxydihydromollugin	C_18_H_20_O_6_	[8]
	49	Epoxymollugin	C_17_H_16_O_5_	[29]
	50	Dihydromollugin	C_17_H_18_O_4_	[7,33]
	51	3-Prenyl-5-methoxynaphthoquinone	C_16_H_16_O_3_	[40]
	52	3-Prenyl-8-methoxynaphthoquinone	C_16_H_16_O_3_	[40]
	53	2-Carbamoyl-3-hydroxynaphthoquinone	C_11_H_7_NO_4_	[36]
	54	2-Carbnmoyl-3-methoxynaphthoquinone	C_12_H_9_NO_4_	[36]
	55	Dehydro-α-lapachone	C_15_H_12_O_3_	[36]
	56	2-Carboxymethyl-3-prenyl-2,3-epoxynaphthoquinone	C_17_H_16_O_5_	[8,29,33,47]
	57	2-Carbomethoxy-3-(3′-hydroxy)isopentyl-1,4-naphthohydroquinone 4-*O*-β-glucoside	C_23_H_30_O_10_	[7]
	58	2-Carbomethoxy-3-prenyl-1,4-naphthohydroquinone 1,4-di-*O*-β-glucoside	C_29_H_38_O_14_	[7]
	59	5-Hydroxy-2-[7-hydroxy-4-(1-hydroxy-1-methylethyl)-2-methyl-6-oxo-2,3,3a,6-tetrahydro-4*H*-1,5-dioxabenzo-[*de*]anthracen-2-yl]-naphtho[1,2-*b*]furan-4-carboxylic acid methyl ester	C_33_H_28_O_9_	[8]
	60	6-Hydroxy-2-(5-hydroxy-4-methoxycarbonyl-naphtho-[1,2-*b*]furan-2-yl)-2-methyl-3,4-dihydro-2*H*-benzo[*h*]-chromene-5-carboxylic acid methyl ester	C_30_H_24_O_8_	[8]
Cyclic hexapeptides	61	RA-I	C_40_H_48_N_6_O_10_	[3,48]
	62	RA-II	C_40_H_48_N_6_O_9_	[48]
	63	RA-III	C_41_H_50_N_6_O_10_	[3,48]
	64	RA-IV	C_41_H_50_N_6_O_10_	[48]
	65	RA-V	C_40_H_48_N_6_O_9_	[3,48]
	66	RA-VI	C_41_H_50_N_6_O_10_	[49]
	67	RA-VII	C_41_H_50_N_6_O_9_	[3,48]
	68	RA-VIII	C_41_H_50_N_6_O_10_	[49]
	69	RA-IX	C_43_H_51_N_6_O_10_	[50]
	70	RA-X	C_43_H_52_N_6_O_11_	[50]
	71	RA-XI	C_42_H_50_N_6_O_11_	[51]
	72	RA-XII	C_46_H_58_N_6_O_14_	[51]
	73	RA-XIII	C_48_H_60_N_6_O_16_	[51]
	74	RA-XIV	C_48_H_58_N_6_O_15_	[51]
	75	RA-XV	C_48_H_60_N_6_O_15_	[52]
	76	RA-XVI	C_48_H_60_N_6_O_16_	[52]
	77	RA-XVII	C_41_H_50_N_6_O_9_	[53]
	78	RA-XVIII	C_41_H_50_N_6_O_10_	[54]
	79	RA-XIX	C_44_H_57_N_6_O_9_	[55]
	80	RA-XX	C_42_H_52_N_6_O_9_	[55]
	81	RA-XXI	C_41_H_50_N_6_O_9_	[55]
	82	RA-XXII	C_41_H_50_N_6_O_10_	[55]
	83	RA-XXIII	C_43_H_53_N_7_O_10_	[56]
	84	RA-XXIV	C_42_H_51_N_7_O_10_	[56]
	85	Rubicordin A	C_46_H_60_N_6_O_14_	[3]
	86	Rubicordin B	C_47_H_62_N_6_O_14_	[3]
	87	Rubicordin C	C_42_H_54_N_6_O_9_	[3]
	88	Rubiyunnanin B	C_46_H_58_N_6_O_14_	[3]
	89	RY-II	C_47_H_60_N_6_O_14_	[3]
	90	neo-RA-V	C_40_H_48_N_6_O_9_	[5]
	91	allo-RA-V	C_40_H_48_N_6_O_9_	[5]
	92	*O*-seco-RA-V	C_40_H_50_N_6_O_9_	[5]
	93	*O*-seco-RA-XXIV	C_42_H_53_N_7_O_10_	[4]
	94	RAI-III	C_41_H_50_N_6_O_10_	[57]
	95	RAI-VI	C_41_H_50_N_6_O_10_	[57]
	96	RA-dimer A	C_80_H_94_N_12_O_18_	[58]
Triterpenoids	97	Oleanolic acid	C_30_H_48_O_3_	[29,33]
	98	Oleanolic aldehyde acetate	C_32_H_50_O_3_	[46]
	99	Rubiarbonol A	C_30_H_50_O_4_	[43,59]
	100	Rubiarbonol B	C_30_H_50_O_3_	[43]
	101	Rubiatriol	C_30_H_50_O_3_	[26]
	102	Rubicoumaric acid	C_39_H_54_O_6_	[10]
	1037	Rubifolic acid	C_30_H_48_O_4_	[10]
	104	Rubiprasin A	C_32_H_52_O_5_	[60]
	105	Rubiprasin B	C_32_H_52_O_4_	[60]
	106	Rubiprasin C	C_32_H_50_O_5_	[60]
	107	Ursolic acid	C_30_H_48_O_3_	[41]
	108	3-β-Friedelinol	C_30_H_52_O_6_	[61]
Other compounds	109	β-Sitostenone	C_29_H_48_O	[43]
	110	β-Sitosterol	C_29_H_50_O	[33,41]
	111	5-Methoxygeniposidic acid	C_17_H_24_O_11_	[62]
	112	6-Methoxygeniposidic acid	C_17_H_24_O_11_	[46]
	113	3,5-di-(*p*-hydroxybenzyl)phenol	C_20_H_18_O_3_	[2]
	114	*n*-Heptadecane	C_17_H_36_	[2]
	115	*n*-Nonadecane	C_19_H_40_	[2]
	116	(+)-Lariciresinol	C_20_H_24_O_6_	[38]
Other compounds	117	3,3′-bis(3,4-Dihydro-4-hydroxy-6-methoxy-2*H*-1-benzopyran)	C_20_H_22_O_6_	[29]
	118	8-Hydroxy *n*-pentadecanyl decan-4-en-1-oate	C_25_H_48_O_3_	[2]
	119	*n*-Octacosanyl octa-1-oate	C_36_H_72_O_2_	[2]
	120	Rubilactone	C_15_H_10_O_5_	[41,45,63]
	121	Rubioncolin B	C_31_H_24_O_10_	[8]
	122	2,3-Dihydro-2-(4-hydroxy-3-methoxyphenyl)-3-hydroxymethyl-5-ω-hydroxypropyl-7-methoxybenzofuran	C_20_H_24_O_6_	[43]
	123	Palmitic acid	C_16_H_32_O_2_	[41]
	124	Tricosanoic acid	C_23_H_46_O_2_	[41]
	125	Rubiasin A	C_15_H_16_O_2_	[38,64]
	126	Rubiasin B	C_15_H_16_O_2_	[64]
	127	Rubiasin C	C_15_H_16_O_2_	[64]
	128	Atraric acid	C_10_H_12_O_4_	[61]
	129	Vanillic acid	C_8_H_8_O_4_	[61]
	130	d-3-*O*-Methoxy-chiroinositol	C_7_H_14_O_6_	[61]
	131	Polysaccharide RPS-1	Not mentioned	[65]
	132	Polysaccharide RPS-2	Not mentioned	[65]
	133	Polysaccharide RPS-3	Not mentioned	[65]
	134	Polysaccharide QA2	Not mentioned	[66]

**Table 2:** Other functions of Rubiae Radix *et* Rhizoma.

Function	Inducer	Test Drug	Model	Efficacy Evaluation	Reference
Anti-adipogenic activity		2-Carboxymethyl-3-prenyl-2,3-epoxynaphthoquinone	3T3-L1 preadipocytes	Induced MMP loss, caspase-3 activation	[54]
				Reduced differentiation-associated accumulation of intracellular lipid	
				Downregulatedexpressions of CCAAT/enhancer binding protein-α, PPAR γ1, PPAR γ2, adiponectin	
Anti-urolithiasis	Ethylene glycol	Ethanol extract	Male Wistar albino rats	Decreased calcium, oxalate levels and number of calcium oxalate crystals deposits in kidney tissue	[90]
Anti-psoriasis		Ethyl acetate fraction of ethanol extract	HaCaT cells	Decreased MMP	[91]
				Induced apoptosis	
			Male BALB/c mice	Increased NGL, TGL and VET	
Anti-nephrotoxicity	Cisplatin	Ethanol extract	Swiss albino mice	Decreased values of serum urea and creatinine	[92]
				Increased GPx, SOD and CAT	
				Inhibited LPO in kidney and liver	
Estrogenic and progestational activity		Ethyl acetate precipitate of methanol extract	Old female albino rats	Increased the regularity of the estrous cycle	[93]
				Increased uterine weight and foetal survival	
Anti-osteoclastogenesis	NF-κB ligand	Mollugin	Mice BMMs	Inhibited osteoclast differentiation	[94]
				Reduced the phosphorylation ofMAP kinase, Akt, and GSK3β	
				Inhibited expression of c-Fos, NFATc1,	
				OSCAR, TRAP, DC-STAMP, OC-STAMP, integrin αν, integrin β3, cathepsin K, and ICAM-1	
Anti-HIV	HIV-1NL4.3	Ethyl acetate extract	CEM-GFP cells	Reduced viral production	[95]

BMMs: bone marrow macrophages; DC-STAMP: dendritic cell-specific transmembrane protein; MMP: mitochondrial membrane potential; NFATc1: nuclear factor of activated T-cells cytoplasmic 1; NGL: number of granular layer; OC-STAMP: osteoclast stimulatory transmembrane protein; OSCAR: osteoclast-associated receptor; PPAR: peroxisome proliferator-activated receptor; TGL: vertical thickness of granular layer; TRAP: tartrate-resistant acid phosphatase; VET: vertical epidermal thickness.
